# The WHO new guideline to control and eliminate human schistosomiasis: implications for the verification of transmission interruption and surveillance of *Schistosoma japonicum* in China

**DOI:** 10.1186/s40249-022-01003-w

**Published:** 2022-07-01

**Authors:** Jing Xu, Shi-Zhu Li, Jia-Gang Guo, Xiao-Nong Zhou, Amadou Garba Djirmay

**Affiliations:** 1 National Institute of Parasitic Diseases, Chinese Center for Disease Control and Prevention (Chinese Center for Tropical Diseases Research), Shanghai, China; 2 NHC Key Laboratory of Parasite and Vector Biology, Shanghai, China; 3 WHO Collaborating Centre for Tropical Diseases, Shanghai, China; 4 National Center for International Research on Tropical Diseases, Shanghai, China; 5 WHO Department of Control of Neglected Tropical Diseases, Geneva, Switzerland

## Introduction

Schistosomiasis is one of the 20 neglected tropical diseases (NTDs) prioritized by World Health Organization (WHO). It is predominately distributed in subtropical and tropical areas of 78 countries and territories with approximate 800 million people at risk of infection and 241.3 million people requiring preventive chemotherapy (PC) [1]*.* Countries are at various stages of control with Africa representing more than 92% of those requiring PC, while Americas and Asia have achieved elimination of schistosomiasis as a public health problem and are moving towards transmission interruption. Due to the increasing available quantity of praziquantel (PZQ) donated by Merck [2] and other non-profit organizations, the treatment coverage for school-aged children (SAC) raised from 25.9% in 2012 to 66.8% by 2019 but declined significantly due to the COVID-19 pandemic [1, 3]. Expansion of PC combined with other interventions resulted in a significant reduction of the prevalence of schistosomiasis including the Africa where 60% prevalence reduction has been observed [4].

To accelerate the achievement of United Nations’ Sustainable Development Goals, WHO published its second roadmap for NTDs in 2021 with specific targets to eliminate schistosomiasis as a public health problem in all endemic countries and interrupt the transmission in selected countries by 2030[5]. To provide guidance for action against human schistosomiasis, WHO launched a new guideline in February 2022 [6]. This article is to discuss in deep the implications of recommendation on verification of transmission interruption with regards to *Schistosoma japonicum* from China perspective.

## Recommendations of the new guideline and approaches to implementation

The new guideline was developed by a guideline development group through a systematic process, including development of the population, intervention, comparator and outcome (PICO) questions formulated by a guideline steering group, followed by systematic reviews, evidence appraisal and synthesis [6]. In accordance with the WHO’s strategy for schistosomiasis control and elimination, six recommendations with implementation remarks based on nine systematic reviews were put forward, which were presented in the additional file ordered by strength of recommendations and certainty of evidence (Additional file 1).

The updated public health strategy in this new guideline encourages member states to implement and integrate into national programmes to control or eliminate schistosomiasis, with following items to be strengthened:

i. As the target population for PC expanded from SAC to all at risk groups, various partners and donors should be ready to fill the gap between current available tablets and requirements in case of increase of PZQ need, and to provide the upcoming pediatric PZQ free of charge for the inclusion of preschool age children in PC. To ensure adequate coverage (≥ 75%) of treatment, PZQ could be delivered alone or in combination with other anti-helminthiasis drugs through school-based or community-based drug delivery systems alone or jointly according to local context. In addition, countries and partners need also to ensure the availability of PZQ for the treatment of infected people at health facilities. This could require integration of PZQ on the list of essential medicine for primary health care units of endemic countries.
ii. Test and treat strategy in low transmission areas using available rapid diagnostic tools, need to be implemented if resources permit. The circulating cathodic antigen (CCA) test or Hematix® dipsticks have shown good performance for screening *S. mansoni* and *S. haematobium* infections respectively, to allow their utilization for test and treat strategy for detection of infected people in communities and basic health centers.
iii. Monitoring and evaluation, and in particular impact assessment surveys are essential for the implementation of the new recommendations. Baseline survey is essential for designing the interventions based on prevalence data, and evaluation survey is needed in areas suspected to be hotspots after two consecutive annual rounds of PC to adjust the frequency of PC. Periodic monitoring and evaluation activities are also needed to assess the treatment coverage, the safety of PZQ in the key age groups as well as the effect of intervention, thus informing the policy-makers to update the strategy of PC.
iv. Multi-sectoral approach to coordinate the Water, Sanitation and Hygiene (WASH) programmes, environmental modification, chemical-based snail control and behavioral change promotion should be strengthened to reduce the transmission and hasten the process of schistosomiasis elimination in areas where applicable. Sharing epidemiological information among sectors will benefit the joint planning of schistosomiasis control programmes and other on-going programmes.
v. In areas approaching the interruption of schistosomiasis transmission, verification framework based on a two-step testing strategy could be applied into national programmes. The choice of testing methods and sampling approach should be considered based on local context and performance of techniques.

## Implication of the guideline for the verification of the transmission interruption in *S. japonicum* areas

With great successes in reducing infection intensity and morbidity associated with schistosomiasis in many countries, recommendation 6 in the new guideline suggests a two-step diagnostic framework starting with a high sensitivity test confirmed with a second, high specificity test for verifying transmission interruption of schistosomiasis which hadn’t been considered in prior WHO guidelines. In China, the two-step diagnostic process had been integrated into national programmes to test *S. japonicum* infection in humans since the World Bank Loan Project was initiated in 1992[7]. Currently, highly sensitive immunological products such as indirect haemagglutation assay, enzyme-linked immunoassay, other rapid diagnostic assays are implemented firstly for screening while only antibody positives are submitted to stool examinations. Kato-Katz thick smear method and miracidia hatching techniques are conducted in parallel to increase the sensitivity of stool examination due to the decline of infection intensity. Individual treatment is delivered to those cases through a test-and-treat approach according to the diagnostic criteria for schistosomiasis, while preventive chemotherapy will be provided to group population at risk of infection due to their water contact behaviors in endemic settings.

Concerning the diagnostics in non-human animals, various modified miracidia hatching techniques are always conducted directly to confirm the infection of schistosomes in domestic animals, but well-developed immunoassays and molecular tools are used limitedly due to the difficulty of collecting blood samples, cross-reaction and higher costs [8, 9]. For intermediate host snails, microscopic dissection or cercaria shedding by exposure to light are still the recommended methods to detect *S. japonicum* infection in snails in routine survey. A loop-mediated isothermal amplification (LAMP) assay has been integrated into national surveillance activities for risk assessment due to its great advantages including high sensitivity and specificity, ease of use and cost-effectiveness when integrated with sample pooling strategy [10]. As there are various molecular techniques developed for *S. japonicum* detection in China, further assessment should be conducted regarding performance, feasibility and cost, to guide the selection of appropriate tools for verification of transmission interruption and surveillance post elimination [9].

## Conclusions

PC remains the main intervention against schistosomiasis but its frequency varies according to the level of prevalence, the history of previous rounds, the impact and the target population in the WHO new guideline. Individuals two years old and above requiring treatment are recommended to be targeted for PC, but the frequency of PC and approach might vary according to the prevalence, the objectives of the programme and the resource available in local settings. Other complementary interventions (WASH, behavior change, snail and animal host control, etc.) are encouraged to accelerate the elimination of schistosomiasis. The diagnostic framework for verifying transmission interruption is recommended but needs development of manuals and standard operating procedures for implementation of the surveys. As the certainty of evidence for six recommendations varied from very low to moderate, the guideline will therefore be updated accordingly as new evidence emerges.

## Supplementary Information

**Additional file 1.** The recommendations of WHO guideline on control and elimination of human schistosomiasis.

## Data Availability

Not applicable.

## Abbreviations

NTDs: Neglected tropical diseases
WHO: World Health Organization
PC: Preventive chemotherapy
PZQ: Praziquantel
PICO: Population, intervention, comparator and outcome
SAC: School-aged children
WASH: Water, sanitation and hygiene
