# Supplementary material for: The WHO new guideline to control and eliminate human schistosomiasis: implications for the verification of transmission interruption and surveillance of Schistosoma japonicum in China
Source: Infect Dis Poverty. 2022 Jul 1;11:79. doi: 10.1186/s40249-022-01003-w (PMC9247933; doi:10.1186/s40249-022-01003-w)
Supplement: Supplementary file 1 — Additional file 1. The recommendations of WHO guideline on control and elimination of human schistosomiasis. [file 40249_2022_1003_MOESM1_ESM.docx]

**Additional file. The recommendations of WHO guideline on control and elimination of human schistosomiasis**

*R1-6 are the serial number of recommendations coded in the WHO guideline, SAC: school-aged children

| **Strength of recommendation** | **Certainty of evidence** | **Recommendations** |
| --- | --- | --- |
| **Strong** | **Moderate** | **R1.**  In endemic communities with prevalence of Schistosoma spp. infection ≥ 10%, WHO recommends annual preventive chemotherapy with a single dose of praziquantel at ≥ 75% treatment coverage in all age groups from 2 years old, including adults, pregnant women after the first trimester and lactating women, to control schistosomiasis morbidity and advance towards eliminating the disease as a public health problem. |
|  |  | **R4.** WHO recommends that health facilities provide access to treatment with praziquantel to control morbidity due to schistosomiasis in all infected individuals regardless of age, including infected pregnant excluding the first trimester, lactating women and pre-SAC aged < 2 years. The decision to administer treatment in children under 2 years of age should be based on testing and clinical judgement. |
|  | **Low** | **R5.** WHO recommends WASH interventions, environmental interventions (water engineering and focal snail control with molluscicides) and behavioural change interventions as essential measures to help reduce transmission of Schistosoma spp. in endemic areas. |
| **Conditional** | **Very low** | **R2.** In endemic communities with prevalence of Schistosoma spp. infection < 10%, WHO suggests one of two approaches based on programmatic objectives and resources: (i) where there has been a programme of regular preventive chemotherapy, to continue the intervention at the same or reduced frequency towards interruption of transmission; or (ii) where there has not been a programme of regular preventive chemotherapy, to use a clinical approach of test-treat, instead of preventive chemotherapy targeting a population. |
|  |  | **R3.** In endemic communities with prevalence of Schistosoma spp. infection ≥ 10% that demonstrate lack of an appropriate response to annual preventive chemotherapy, despite adequate treatment coverage (≥ 75%), WHO suggests consideration of biannual (twice yearly) instead of annual preventive chemotherapy. |
|  |  | **R6.** In communities approaching the interruption of transmission (defined as having no autochthonous human cases reported for 5 consecutive years), WHO suggests a verification framework that consists of:1. Testing for Schistosoma infection in humans with a diagnostic that has high sensitivity and specificity. This may require the use of a two-step diagnostic process starting with a high sensitivity test confirmed with a second, high specificity test. 2. Testing for Schistosoma infection in snails with a diagnostic that has high sensitivity and specificity. This may require the use of a two-step diagnostic process starting with a high sensitivity test confirmed with a second, high specificity test. 3. Testing for Schistosoma infection in non-human mammalian hosts, as applicable, with a diagnostic that has high sensitivity and specificity. This may require the use of a two-step diagnostic process starting with a high sensitivity test confirmed with a second, high specificity test. |
